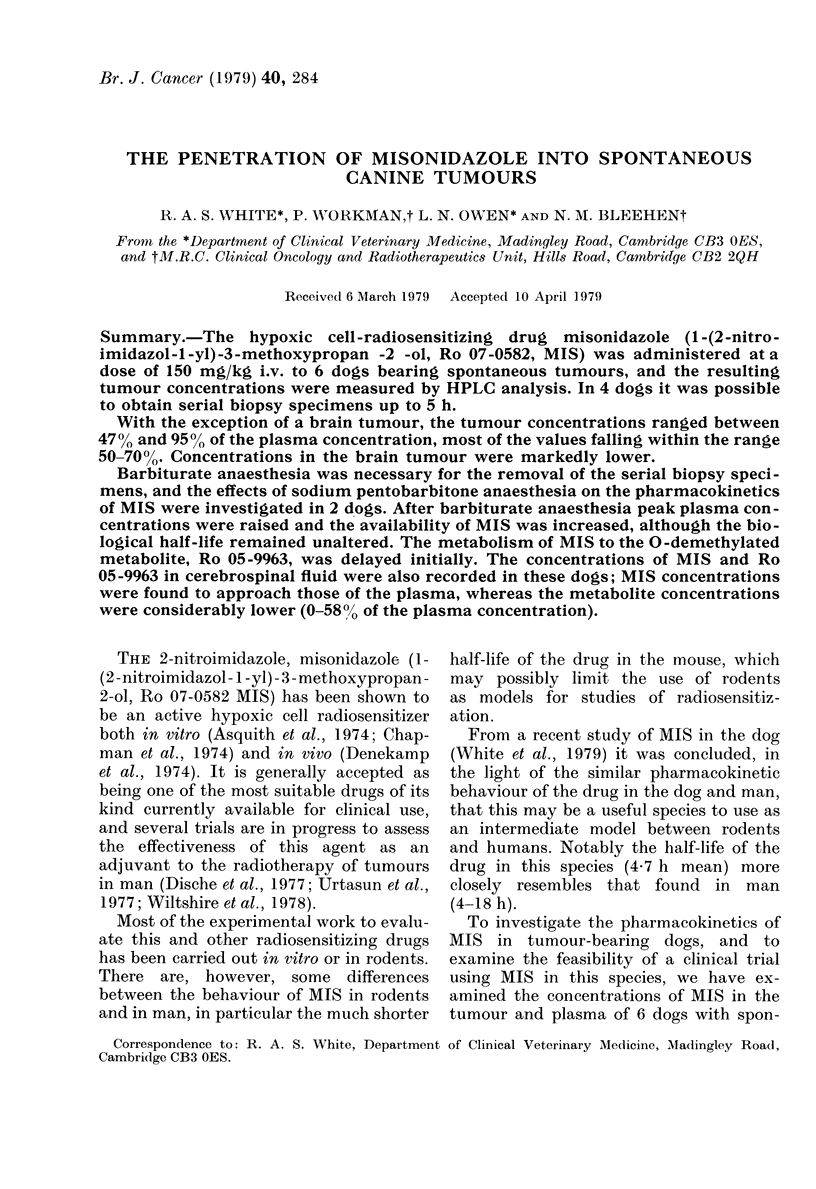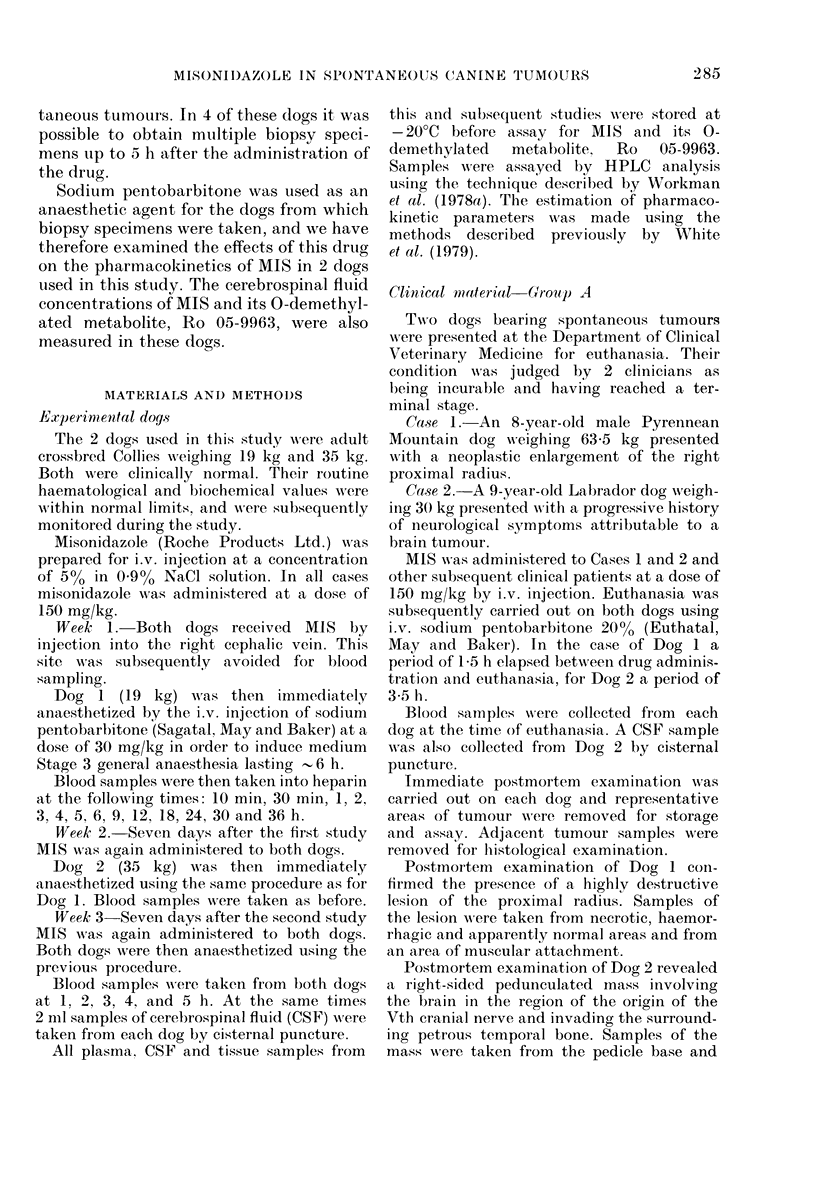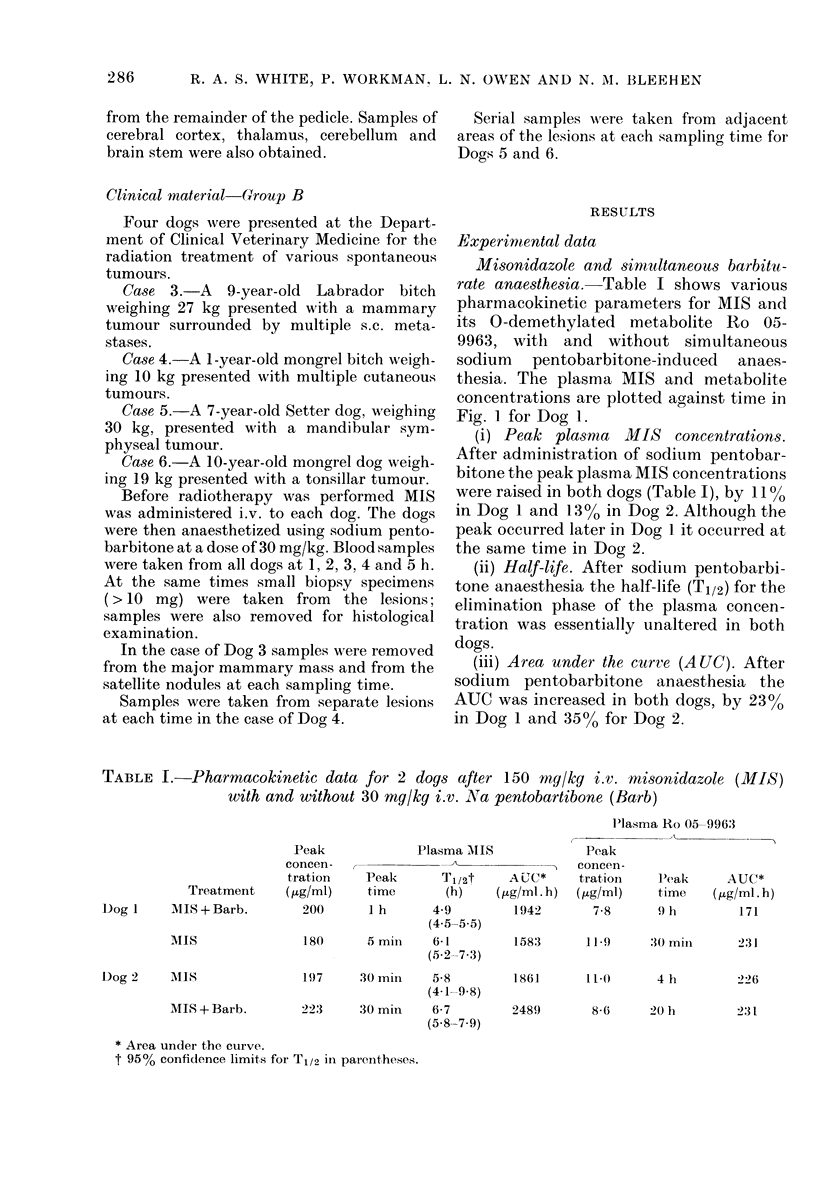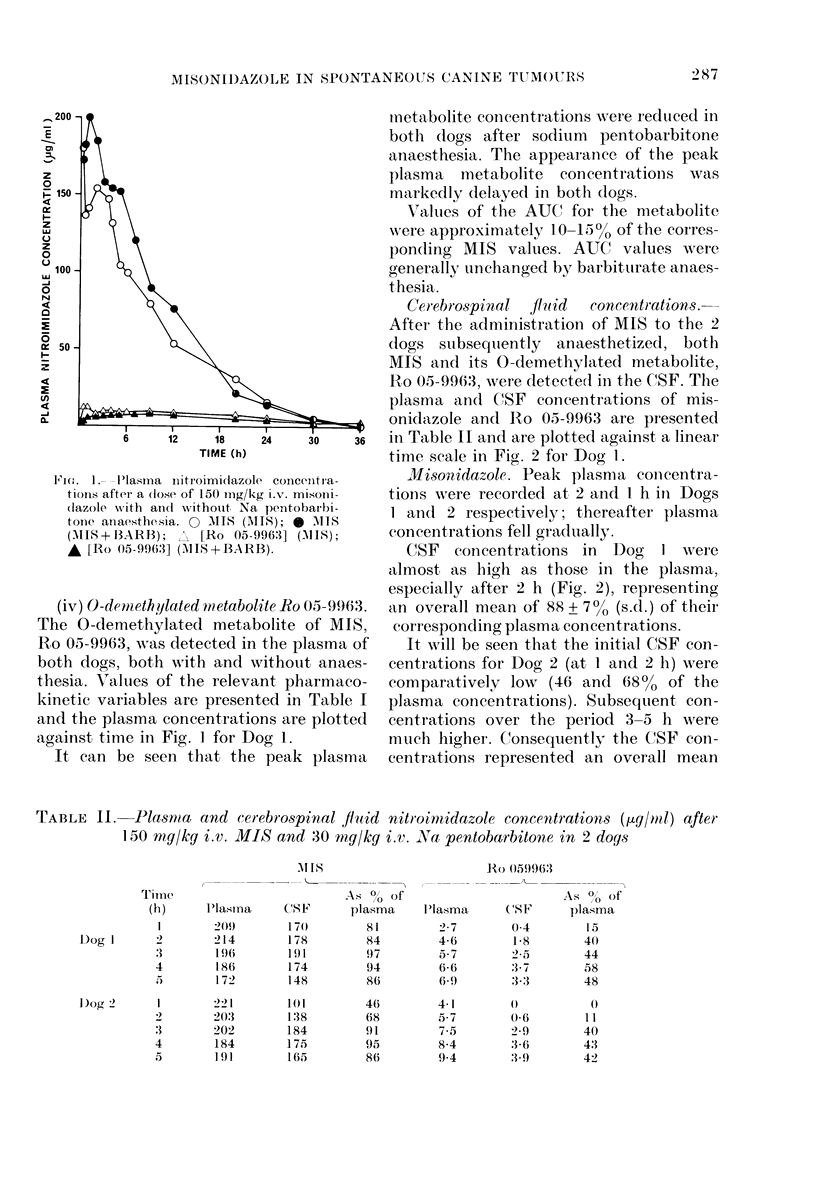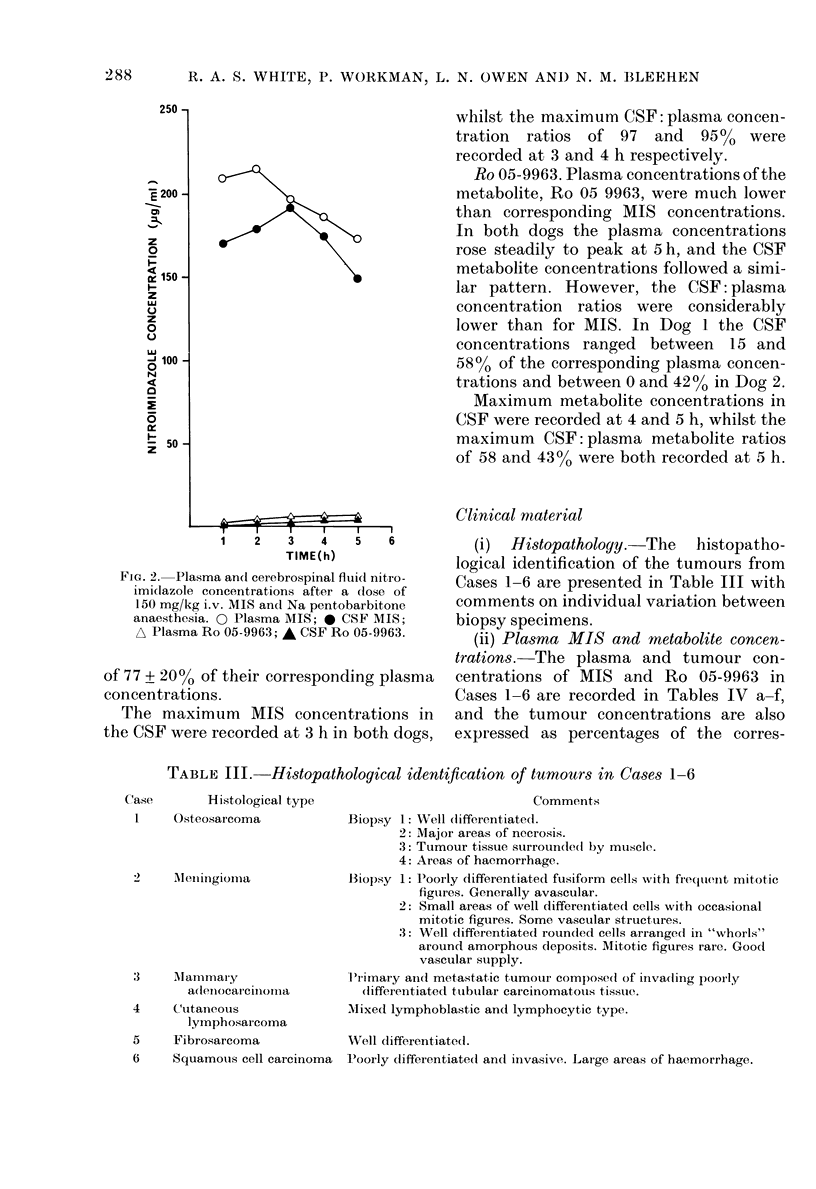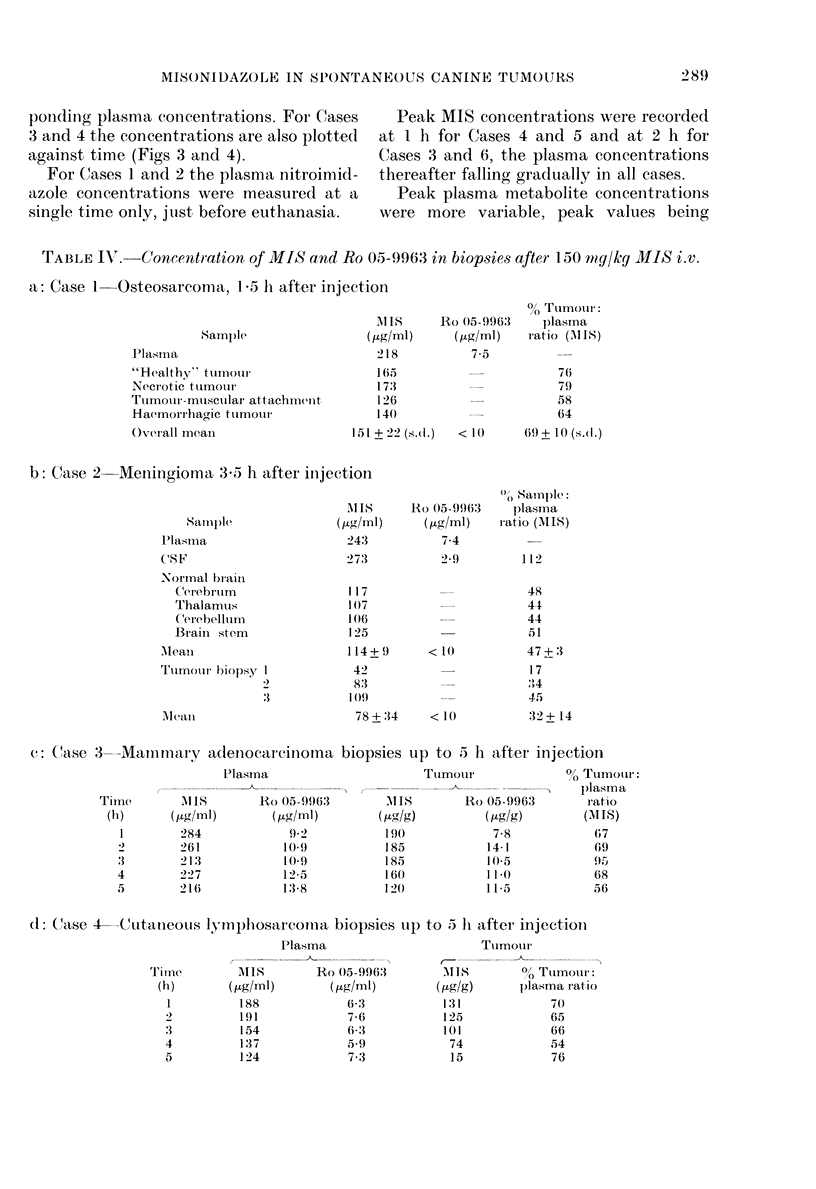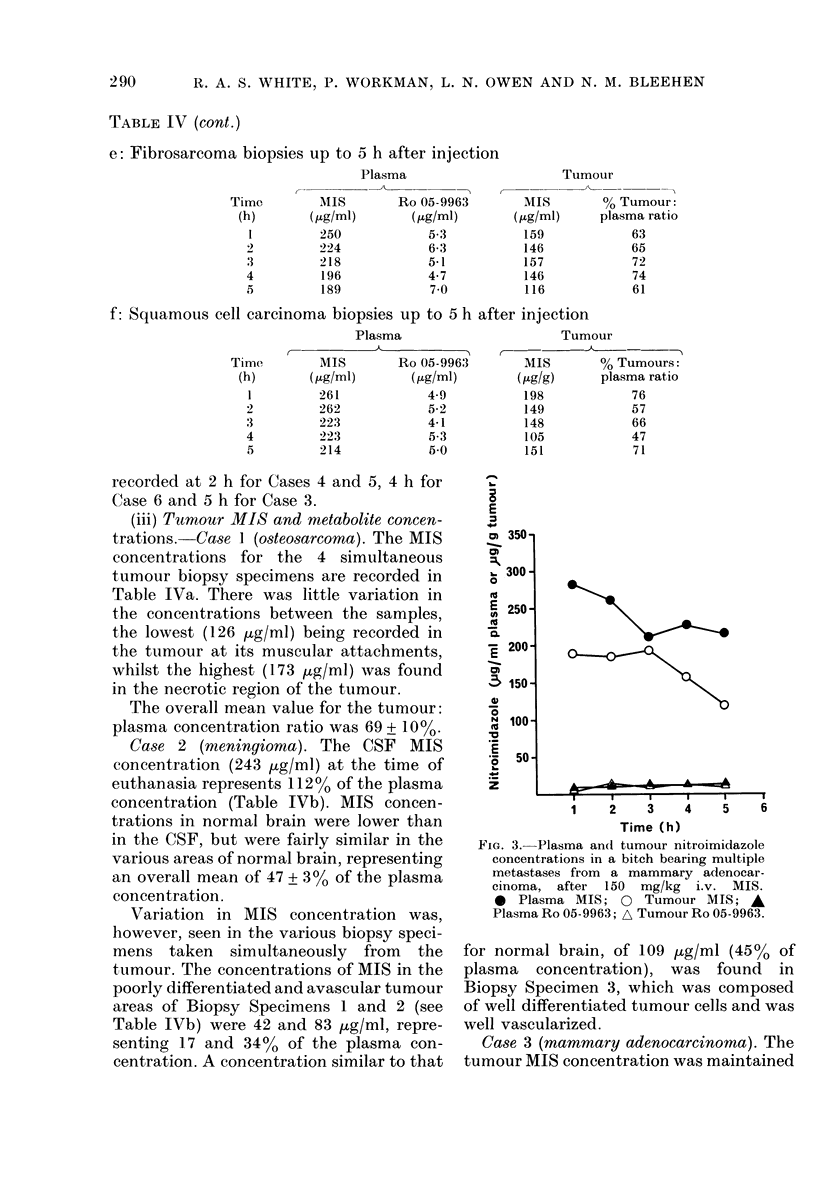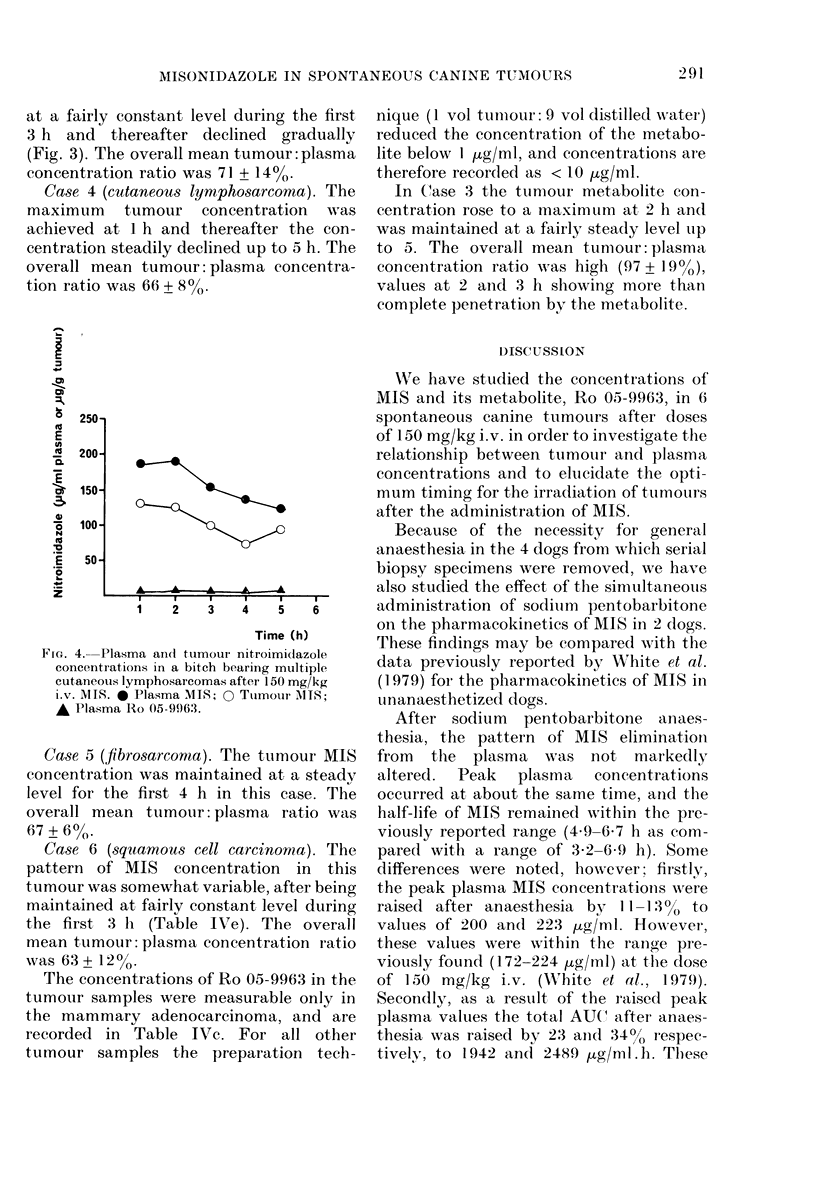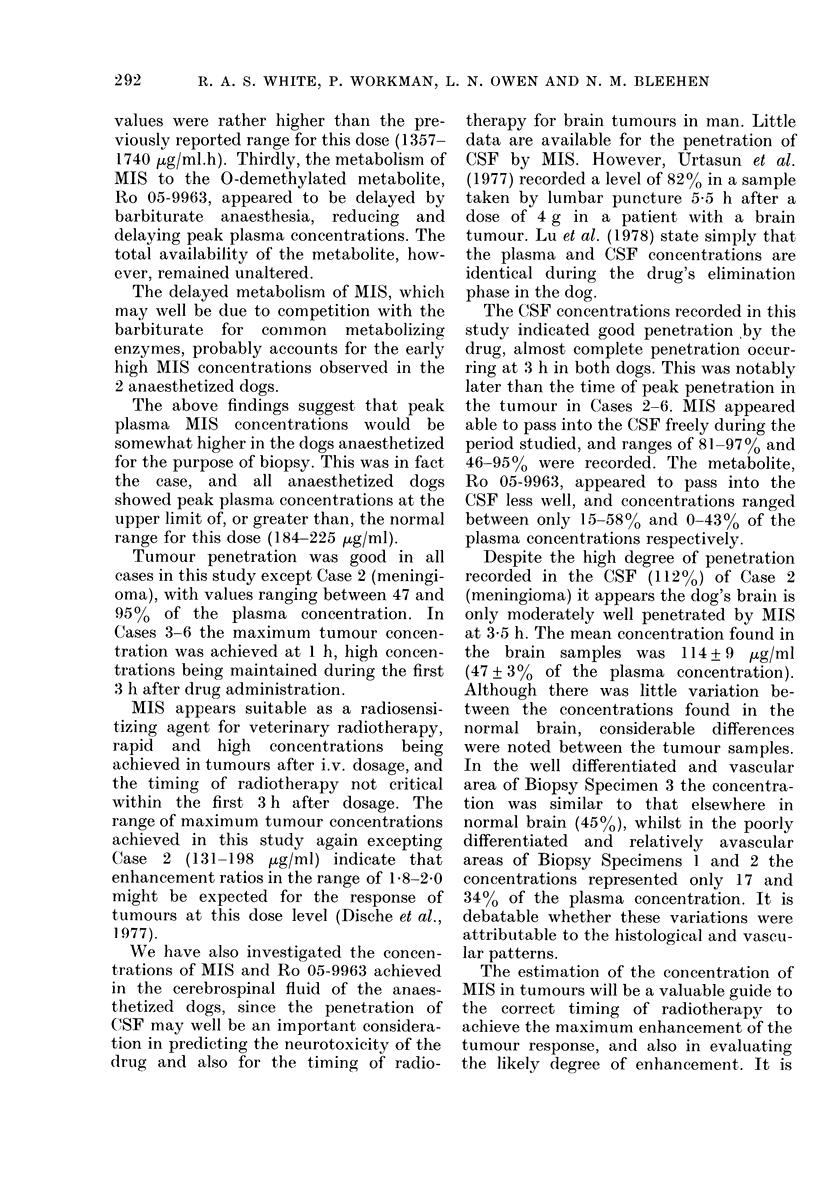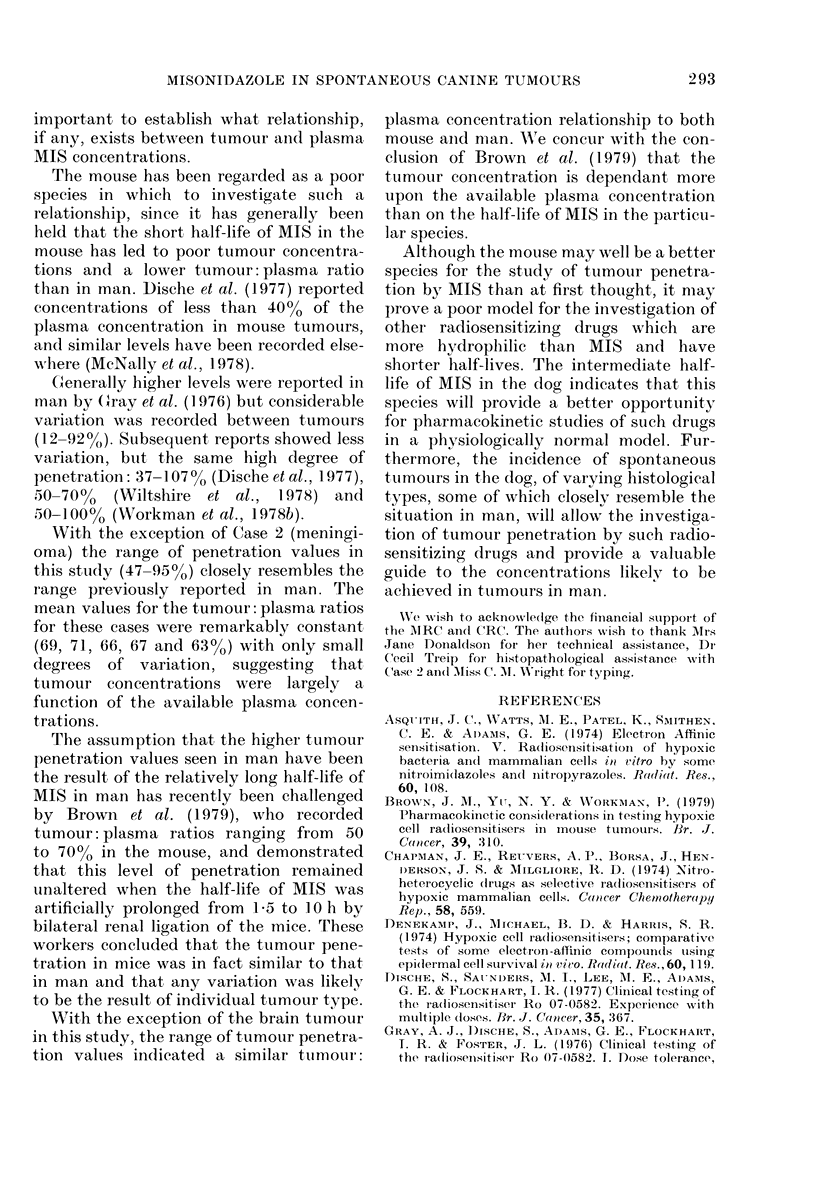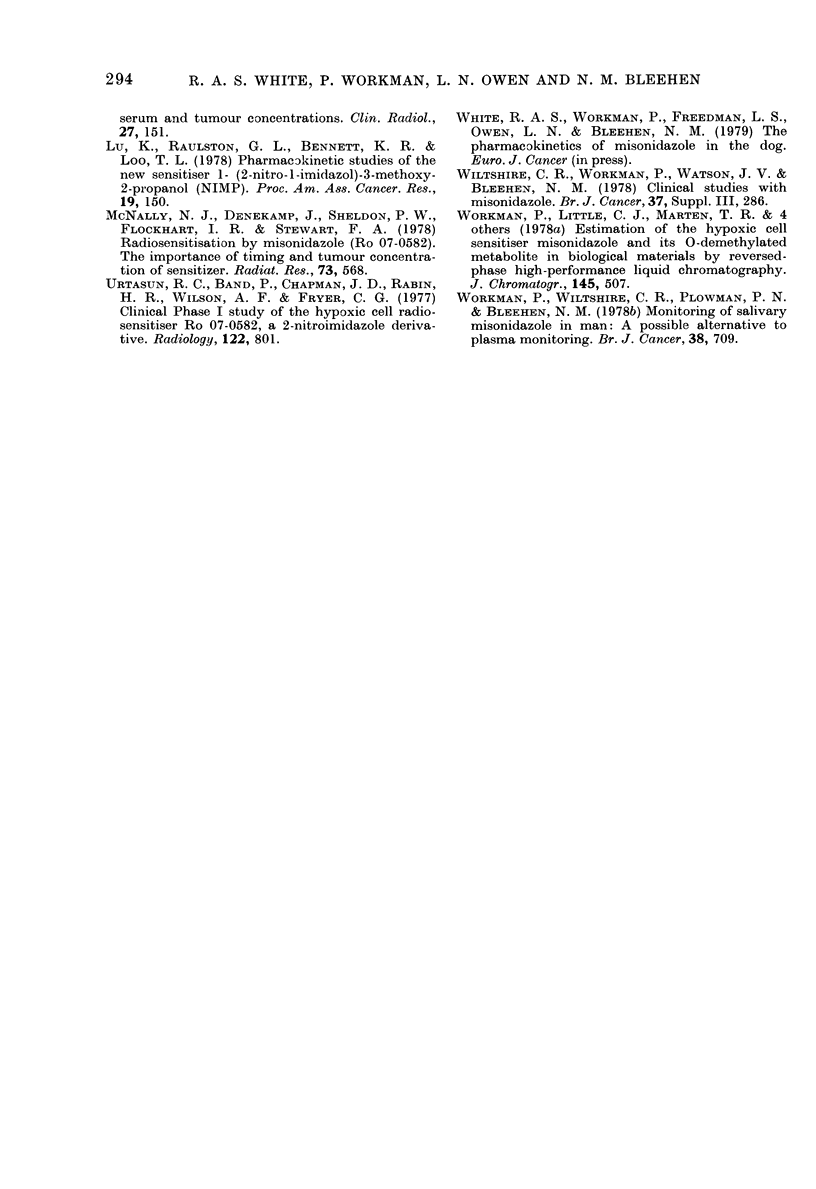# The penetration of misonidazole into spontaneous canine tumours.

**DOI:** 10.1038/bjc.1979.177

**Published:** 1979-08

**Authors:** R. A. White, P. Workman, L. N. Owen, N. M. Bleehen

## Abstract

The hypoxic cell-radiosensitizing drug misonidazole (1-(2-nitroimidazol-1-yl)-3-methoxypropan -2 -ol, Ro 07-0582, MIS) was administered at a dose of 150 mg/kg i.v. to 6 dogs bearing spontaneous tumours, and the resulting tumour concentrations were measured to HPLC analysis. In 4 dogs it was possible to obtain serial biopsy specimens up to 5 h. With the exception of a brain tumour, the tumour concentrations ranged between 47% and 95% of the plasma concentration, most of the values falling within the range 50--70%. Concentrations in the brain tumour were markedly lower. Barbiturate anaesthesia was necessary for the removal of the serial biopsy specimens, and the effects of sodium pentobarbitone anaesthesia on the pharmacokinetics of MIS were investigated in 2 dogs. After barbiturate anaesthesia peak plasma concontrations were raised and the availability of MIS was increased, although the biological half-life remained unaltered. The metabolism of MIS to the O-demethylated metabolite, Ro 05-9963, was delayed initially. The concentrations of MIS AND Ro 05-9963 in cerebrospinal fluid were also recorded in these dogs; MIS concentrations were found to approach those of the plasma, whereas the metabolite concentrations were considerably lower (0--58% of the plasma concentration).


*Br. J. Cancer* (1979) **40**, 284

### THE PENETRATION OF MISONIDAZOLE INTO SPONTANEOUS CANINE TUMOURS

R. A. S. WHITE*, P. WORKMAN,† L. N. OWEN* AND N. M. BLEEHEN†

*From the *Department of Clinical Veterinary Medicine, Madingley Road, Cambridge CB3 0ES, and †M.R.C. Clinical Oncology and Radiotherapeutics Unit, Hills Road, Cambridge CB2 2QH*

Received 6 March 1979 Accepted 10 April 1979

**Summary.—The hypoxic cell-radiosensitizing drug misonidazole (1-(2-nitro-imidazol-1-yl)-3-methoxypropan -2 -ol, Ro 07-0582, MIS) was administered at a dose of 150 mg/kg i.v. to 6 dogs bearing spontaneous tumours, and the resulting tumour concentrations were measured by HPLC analysis. In 4 dogs it was possible to obtain serial biopsy specimens up to 5 h.**

**With the exception of a brain tumour, the tumour concentrations ranged between 47% and 95% of the plasma concentration, most of the values falling within the range 50–70%. Concentrations in the brain tumour were markedly lower.**

**Barbiturate anaesthesia was necessary for the removal of the serial biopsy specimens, and the effects of sodium pentobarbitone anaesthesia on the pharmacokinetics of MIS were investigated in 2 dogs. After barbiturate anaesthesia peak plasma concentrations were raised and the availability of MIS was increased, although the biological half-life remained unaltered. The metabolism of MIS to the O-demethylated metabolite, Ro 05-9963, was delayed initially. The concentrations of MIS and Ro 05-9963 in cerebrospinal fluid were also recorded in these dogs; MIS concentrations were found to approach those of the plasma, whereas the metabolite concentrations were considerably lower (0–58% of the plasma concentration).**

THE 2-nitroimidazole, misonidazole (1-(2-nitroimidazol-1-yl)-3-methoxypropan-2-ol, Ro 07-0582 MIS) has been shown to be an active hypoxic cell radiosensitizer both *in vitro* (Asquith *et al.*, 1974; Chapman *et al.*, 1974) and *in vivo* (Denekamp *et al.*, 1974). It is generally accepted as being one of the most suitable drugs of its kind currently available for clinical use, and several trials are in progress to assess the effectiveness of this agent as an adjuvant to the radiotherapy of tumours in man (Dische *et al.*, 1977; Urtasun *et al.*, 1977; Wiltshire *et al.*, 1978).

Most of the experimental work to evaluate this and other radiosensitizing drugs has been carried out *in vitro* or in rodents. There are, however, some differences between the behaviour of MIS in rodents and in man, in particular the much shorter half-life of the drug in the mouse, which may possibly limit the use of rodents as models for studies of radiosensitization.

From a recent study of MIS in the dog (White *et al.*, 1979) it was concluded, in the light of the similar pharmacokinetic behaviour of the drug in the dog and man, that this may be a useful species to use as an intermediate model between rodents and humans. Notably the half-life of the drug in this species (4·7 h mean) more closely resembles that found in man (4–18 h).

To investigate the pharmacokinetics of MIS in tumour-bearing dogs, and to examine the feasibility of a clinical trial using MIS in this species, we have examined the concentrations of MIS in the tumour and plasma of 6 dogs with spon-

Correspondence to: R. A. S. White, Department of Clinical Veterinary Medicine, Madingley Road, Cambridge CB3 0ES.

taneous tumours. In 4 of these dogs it was possible to obtain multiple biopsy specimens up to 5 h after the administration of the drug.

Sodium pentobarbitone was used as an anaesthetic agent for the dogs from which biopsy specimens were taken, and we have therefore examined the effects of this drug on the pharmacokinetics of MIS in 2 dogs used in this study. The cerebrospinal fluid concentrations of MIS and its O-demethylated metabolite, Ro 05-9963, were also measured in these dogs.

### MATERIALS AND METHODS

*Experimental dogs*

The 2 dogs used in this study were adult crossbred Collies weighing 19 kg and 35 kg. Both were clinically normal. Their routine haematological and biochemical values were within normal limits, and were subsequently monitored during the study.

Misonidazole (Roche Products Ltd.) was prepared for i.v. injection at a concentration of 5% in 0·9% NaCl solution. In all cases misonidazole was administered at a dose of 150 mg/kg.

*Week* 1.—Both dogs received MIS by injection into the right cephalic vein. This site was subsequently avoided for blood sampling.

Dog 1 (19 kg) was then immediately anaesthetized by the i.v. injection of sodium pentobarbitone (Sagatal, May and Baker) at a dose of 30 mg/kg in order to induce medium Stage 3 general anaesthesia lasting ~6 h.

Blood samples were then taken into heparin at the following times: 10 min, 30 min, 1, 2, 3, 4, 5, 6, 9, 12, 18, 24, 30 and 36 h.

*Week* 2.—Seven days after the first study MIS was again administered to both dogs.

Dog 2 (35 kg) was then immediately anaesthetized using the same procedure as for Dog 1. Blood samples were taken as before.

*Week* 3—Seven days after the second study MIS was again administered to both dogs. Both dogs were then anaesthetized using the previous procedure.

Blood samples were taken from both dogs at 1, 2, 3, 4, and 5 h. At the same times 2 ml samples of cerebrospinal fluid (CSF) were taken from each dog by cisternal puncture.

All plasma, CSF and tissue samples from this and subsequent studies were stored at −20°C before assay for MIS and its O-demethylated metabolite, Ro 05-9963. Samples were assayed by HPLC analysis using the technique described by Workman *et al.* (1978*a*). The estimation of pharmacokinetic parameters was made using the methods described previously by White *et al.* (1979).

*Clinical material—Group A*

Two dogs bearing spontaneous tumours were presented at the Department of Clinical Veterinary Medicine for euthanasia. Their condition was judged by 2 clinicians as being incurable and having reached a terminal stage.

*Case* 1.—An 8-year-old male Pyrennean Mountain dog weighing 63·5 kg presented with a neoplastic enlargement of the right proximal radius.

*Case* 2.—A 9-year-old Labrador dog weighing 30 kg presented with a progressive history of neurological symptoms attributable to a brain tumour.

MIS was administered to Cases 1 and 2 and other subsequent clinical patients at a dose of 150 mg/kg by i.v. injection. Euthanasia was subsequently carried out on both dogs using i.v. sodium pentobarbitone 20% (Euthatal, May and Baker). In the case of Dog 1 a period of 1·5 h elapsed between drug administration and euthanasia, for Dog 2 a period of 3·5 h.

Blood samples were collected from each dog at the time of euthanasia. A CSF sample was also collected from Dog 2 by cisternal puncture.

Immediate postmortem examination was carried out on each dog and representative areas of tumour were removed for storage and assay. Adjacent tumour samples were removed for histological examination.

Postmortem examination of Dog 1 confirmed the presence of a highly destructive lesion of the proximal radius. Samples of the lesion were taken from necrotic, haemorrhagic and apparently normal areas and from an area of muscular attachment.

Postmortem examination of Dog 2 revealed a right-sided pedunculated mass involving the brain in the region of the origin of the Vth cranial nerve and invading the surrounding petrous temporal bone. Samples of the mass were taken from the pedicle base and from the remainder of the pedicle. Samples of cerebral cortex, thalamus, cerebellum and brain stem were also obtained.

*Clinical material—Group B*

Four dogs were presented at the Department of Clinical Veterinary Medicine for the radiation treatment of various spontaneous tumours.

*Case* 3.—A 9-year-old Labrador bitch weighing 27 kg presented with a mammary tumour surrounded by multiple s.c. metastases.

*Case* 4.—A 1-year-old mongrel bitch weighing 10 kg presented with multiple cutaneous tumours.

*Case* 5.—A 7-year-old Setter dog, weighing 30 kg, presented with a mandibular symphyseal tumour.

*Case* 6.—A 10-year-old mongrel dog weighing 19 kg presented with a tonsillar tumour.

Before radiotherapy was performed MIS was administered i.v. to each dog. The dogs were then anaesthetized using sodium pentobarbitone at a dose of 30 mg/kg. Blood samples were taken from all dogs at 1, 2, 3, 4 and 5 h. At the same times small biopsy specimens (>10 mg) were taken from the lesions; samples were also removed for histological examination.

In the case of Dog 3 samples were removed from the major mammary mass and from the satellite nodules at each sampling time.

Samples were taken from separate lesions at each time in the case of Dog 4.

Serial samples were taken from adjacent areas of the lesions at each sampling time for Dogs 5 and 6.

### RESULTS

*Experimental data*

*Misonidazole and simultaneous barbiturate anaesthesia.*—Table I shows various pharmacokinetic parameters for MIS and its O-demethylated metabolite Ro 05-9963, with and without simultaneous sodium pentobarbitone-induced anaesthesia. The plasma MIS and metabolite concentrations are plotted against time in Fig. 1 for Dog 1.

(i) *Peak plasma MIS concentrations.* After administration of sodium pentobarbitone the peak plasma MIS concentrations were raised in both dogs (Table I), by 11% in Dog 1 and 13% in Dog 2. Although the peak occurred later in Dog 1 it occurred at the same time in Dog 2.

(ii) *Half-life.* After sodium pentobarbitone anaesthesia the half-life ($T_{1/2}$) for the elimination phase of the plasma concentration was essentially unaltered in both dogs.

(iii) *Area under the curve (AUC).* After sodium pentobarbitone anaesthesia the AUC was increased in both dogs, by 23% in Dog 1 and 35% for Dog 2.

TABLE I.—*Pharmacokinetic data for* 2 *dogs after* 150 *mg/kg i.v. misonidazole (MIS) with and without* 30 *mg/kg i.v. Na pentobartibone (Barb)*

| | | Plasma MIS | | | | Plasma Ro 05-9963 | | |
|---|---|---|---|---|---|---|---|---|
| | Treatment | Peak concentration (μg/ml) | Peak time | $T_{1/2}$† (h) | AUC* (μg/ml.h) | Peak concentration (μg/ml) | Peak time | AUC* (μg/ml.h) |
| Dog 1 | MIS + Barb. | 200 | 1 h | 4·9 (4·5–5·5) | 1942 | 7·8 | 9 h | 171 |
| | MIS | 180 | 5 min | 6·1 (5·2–7·3) | 1583 | 11·9 | 30 min | 231 |
| Dog 2 | MIS | 197 | 30 min | 5·8 (4·1–9·8) | 1861 | 11·0 | 4 h | 226 |
| | MIS + Barb. | 223 | 30 min | 6·7 (5·8–7·9) | 2489 | 8·6 | 20 h | 231 |

* Area under the curve.
† 95% confidence limits for $T_{1/2}$ in parentheses.

FIG. 1.—Plasma nitroimidazole concentrations after a dose of 150 mg/kg i.v. misonidazole with and without Na pentobarbitone anaesthesia. ○ MIS (MIS); ● MIS (MIS+BARB); △ [Ro 05-9963] (MIS); ▲ [Ro 05-9963] (MIS+BARB).

(iv) *O-demethylated metabolite Ro* 05-9963. The O-demethylated metabolite of MIS, Ro 05-9963, was detected in the plasma of both dogs, both with and without anaesthesia. Values of the relevant pharmacokinetic variables are presented in Table I and the plasma concentrations are plotted against time in Fig. 1 for Dog 1.

It can be seen that the peak plasma metabolite concentrations were reduced in both dogs after sodium pentobarbitone anaesthesia. The appearance of the peak plasma metabolite concentrations was markedly delayed in both dogs.

Values of the AUC for the metabolite were approximately 10–15% of the corresponding MIS values. AUC values were generally unchanged by barbiturate anaesthesia.

*Cerebrospinal fluid concentrations.*—After the administration of MIS to the 2 dogs subsequently anaesthetized, both MIS and its O-demethylated metabolite, Ro 05-9963, were detected in the CSF. The plasma and CSF concentrations of misonidazole and Ro 05-9963 are presented in Table II and are plotted against a linear time scale in Fig. 2 for Dog 1.

*Misonidazole.* Peak plasma concentrations were recorded at 2 and 1 h in Dogs 1 and 2 respectively; thereafter plasma concentrations fell gradually.

CSF concentrations in Dog 1 were almost as high as those in the plasma, especially after 2 h (Fig. 2), representing an overall mean of 88 ± 7% (s.d.) of their corresponding plasma concentrations.

It will be seen that the initial CSF concentrations for Dog 2 (at 1 and 2 h) were comparatively low (46 and 68% of the plasma concentrations). Subsequent concentrations over the period 3–5 h were much higher. Consequently the CSF concentrations represented an overall mean

TABLE II.—*Plasma and cerebrospinal fluid nitroimidazole concentrations (μg/ml) after 150 mg/kg i.v. MIS and 30 mg/kg i.v. Na pentobarbitone in 2 dogs*

| | | MIS | | | Ro 059963 | | |
|---|---|---|---|---|---|---|---|
| | Time (h) | Plasma | CSF | As % of plasma | Plasma | CSF | As % of plasma |
| | 1 | 209 | 170 | 81 | 2·7 | 0·4 | 15 |
| Dog 1 | 2 | 214 | 178 | 84 | 4·6 | 1·8 | 40 |
| | 3 | 196 | 191 | 97 | 5·7 | 2·5 | 44 |
| | 4 | 186 | 174 | 94 | 6·6 | 3·7 | 58 |
| | 5 | 172 | 148 | 86 | 6·9 | 3·3 | 48 |
| Dog 2 | 1 | 221 | 101 | 46 | 4·1 | 0 | 0 |
| | 2 | 203 | 138 | 68 | 5·7 | 0·6 | 11 |
| | 3 | 202 | 184 | 91 | 7·5 | 2·9 | 40 |
| | 4 | 184 | 175 | 95 | 8·4 | 3·6 | 43 |
| | 5 | 191 | 165 | 86 | 9·4 | 3·9 | 42 |

FIG. 2.—Plasma and cerebrospinal fluid nitroimidazole concentrations after a dose of 150 mg/kg i.v. MIS and Na pentobarbitone anaesthesia. ○ Plasma MIS; ● CSF MIS; △ Plasma Ro 05-9963; ▲ CSF Ro 05-9963.

of 77 ± 20% of their corresponding plasma concentrations.

The maximum MIS concentrations in the CSF were recorded at 3 h in both dogs, whilst the maximum CSF: plasma concentration ratios of 97 and 95% were recorded at 3 and 4 h respectively.

*Ro* 05-9963. Plasma concentrations of the metabolite, Ro 05 9963, were much lower than corresponding MIS concentrations. In both dogs the plasma concentrations rose steadily to peak at 5 h, and the CSF metabolite concentrations followed a similar pattern. However, the CSF: plasma concentration ratios were considerably lower than for MIS. In Dog 1 the CSF concentrations ranged between 15 and 58% of the corresponding plasma concentrations and between 0 and 42% in Dog 2.

Maximum metabolite concentrations in CSF were recorded at 4 and 5 h, whilst the maximum CSF: plasma metabolite ratios of 58 and 43% were both recorded at 5 h.

### *Clinical material*

(i) *Histopathology.*—The histopathological identification of the tumours from Cases 1–6 are presented in Table III with comments on individual variation between biopsy specimens.

(ii) *Plasma MIS and metabolite concentrations.*—The plasma and tumour concentrations of MIS and Ro 05-9963 in Cases 1–6 are recorded in Tables IV a–f, and the tumour concentrations are also expressed as percentages of the corres-

TABLE III.—*Histopathological identification of tumours in Cases* 1–6

| Case | Histological type | Comments |
|---|---|---|
| 1 | Osteosarcoma | Biopsy 1: Well differentiated.<br>2: Major areas of necrosis.<br>3: Tumour tissue surrounded by muscle.<br>4: Areas of haemorrhage. |
| 2 | Meningioma | Biopsy 1: Poorly differentiated fusiform cells with frequent mitotic figures. Generally avascular.<br>2: Small areas of well differentiated cells with occasional mitotic figures. Some vascular structures.<br>3: Well differentiated rounded cells arranged in "whorls" around amorphous deposits. Mitotic figures rare. Good vascular supply. |
| 3 | Mammary adenocarcinoma | Primary and metastatic tumour composed of invading poorly differentiated tubular carcinomatous tissue. |
| 4 | Cutaneous lymphosarcoma | Mixed lymphoblastic and lymphocytic type. |
| 5 | Fibrosarcoma | Well differentiated. |
| 6 | Squamous cell carcinoma | Poorly differentiated and invasive. Large areas of haemorrhage. |

ponding plasma concentrations. For Cases 3 and 4 the concentrations are also plotted against time (Figs 3 and 4).

For Cases 1 and 2 the plasma nitroimidazole concentrations were measured at a single time only, just before euthanasia.

Peak MIS concentrations were recorded at 1 h for Cases 4 and 5 and at 2 h for Cases 3 and 6, the plasma concentrations thereafter falling gradually in all cases.

Peak plasma metabolite concentrations were more variable, peak values being

TABLE IV.—*Concentration of MIS and Ro* 05-9963 *in biopsies after* 150 *mg/kg MIS i.v.*

a: Case 1—Osteosarcoma, 1·5 h after injection

| Sample | MIS (μg/ml) | Ro 05-9963 (μg/ml) | % Tumour: plasma ratio (MIS) |
|---|---|---|---|
| Plasma | 218 | 7·5 | — |
| "Healthy" tumour | 165 | — | 76 |
| Necrotic tumour | 173 | — | 79 |
| Tumour-muscular attachment | 126 | — | 58 |
| Haemorrhagic tumour | 140 | — | 64 |
| Overall mean | 151 ± 22 (s.d.) | < 10 | 69 ± 10 (s.d.) |

b: Case 2—Meningioma 3·5 h after injection

| Sample | MIS (μg/ml) | Ro 05-9963 (μg/ml) | % Sample: plasma ratio (MIS) |
|---|---|---|---|
| Plasma | 243 | 7·4 | — |
| CSF | 273 | 2·9 | 112 |
| Normal brain | | | |
| Cerebrum | 117 | — | 48 |
| Thalamus | 107 | — | 44 |
| Cerebellum | 106 | — | 44 |
| Brain stem | 125 | — | 51 |
| Mean | 114 ± 9 | < 10 | 47 ± 3 |
| Tumour biopsy 1 | 42 | — | 17 |
| 2 | 83 | — | 34 |
| 3 | 109 | — | 45 |
| Mean | 78 ± 34 | < 10 | 32 ± 14 |

c: Case 3—Mammary adenocarcinoma biopsies up to 5 h after injection

| Time (h) | Plasma MIS (μg/ml) | Plasma Ro 05-9963 (μg/ml) | Tumour MIS (μg/g) | Tumour Ro 05-9963 (μg/g) | % Tumour: plasma ratio (MIS) |
|---|---|---|---|---|---|
| 1 | 284 | 9·2 | 190 | 7·8 | 67 |
| 2 | 261 | 10·9 | 185 | 14·1 | 69 |
| 3 | 213 | 10·9 | 185 | 10·5 | 95 |
| 4 | 227 | 12·5 | 160 | 11·0 | 68 |
| 5 | 216 | 13·8 | 120 | 11·5 | 56 |

d: Case 4—Cutaneous lymphosarcoma biopsies up to 5 h after injection

| Time (h) | Plasma MIS (μg/ml) | Plasma Ro 05-9963 (μg/ml) | Tumour MIS (μg/g) | Tumour % Tumour: plasma ratio |
|---|---|---|---|---|
| 1 | 188 | 6·3 | 131 | 70 |
| 2 | 191 | 7·6 | 125 | 65 |
| 3 | 154 | 6·3 | 101 | 66 |
| 4 | 137 | 5·9 | 74 | 54 |
| 5 | 124 | 7·3 | 15 | 76 |

TABLE IV (*cont.*)

e: Fibrosarcoma biopsies up to 5 h after injection

| Time (h) | Plasma MIS ($\mu$g/ml) | Plasma Ro 05-9963 ($\mu$g/ml) | Tumour MIS ($\mu$g/ml) | Tumour % Tumour: plasma ratio |
|---|---|---|---|---|
| 1 | 250 | 5·3 | 159 | 63 |
| 2 | 224 | 6·3 | 146 | 65 |
| 3 | 218 | 5·1 | 157 | 72 |
| 4 | 196 | 4·7 | 146 | 74 |
| 5 | 189 | 7·0 | 116 | 61 |

f: Squamous cell carcinoma biopsies up to 5 h after injection

| Time (h) | Plasma MIS ($\mu$g/ml) | Plasma Ro 05-9963 ($\mu$g/ml) | Tumour MIS ($\mu$g/g) | Tumour % Tumours: plasma ratio |
|---|---|---|---|---|
| 1 | 261 | 4·9 | 198 | 76 |
| 2 | 262 | 5·2 | 149 | 57 |
| 3 | 223 | 4·1 | 148 | 66 |
| 4 | 223 | 5·3 | 105 | 47 |
| 5 | 214 | 5·0 | 151 | 71 |

recorded at 2 h for Cases 4 and 5, 4 h for Case 6 and 5 h for Case 3.

(iii) *Tumour MIS and metabolite concentrations.*—*Case* 1 (*osteosarcoma*). The MIS concentrations for the 4 simultaneous tumour biopsy specimens are recorded in Table IVa. There was little variation in the concentrations between the samples, the lowest (126 $\mu$g/ml) being recorded in the tumour at its muscular attachments, whilst the highest (173 $\mu$g/ml) was found in the necrotic region of the tumour.

The overall mean value for the tumour: plasma concentration ratio was $69 \pm 10\%$.

*Case* 2 (*meningioma*). The CSF MIS concentration (243 $\mu$g/ml) at the time of euthanasia represents 112% of the plasma concentration (Table IVb). MIS concentrations in normal brain were lower than in the CSF, but were fairly similar in the various areas of normal brain, representing an overall mean of $47 \pm 3\%$ of the plasma concentration.

Variation in MIS concentration was, however, seen in the various biopsy specimens taken simultaneously from the tumour. The concentrations of MIS in the poorly differentiated and avascular tumour areas of Biopsy Specimens 1 and 2 (see Table IVb) were 42 and 83 $\mu$g/ml, representing 17 and 34% of the plasma concentration. A concentration similar to that

FIG. 3.—Plasma and tumour nitroimidazole concentrations in a bitch bearing multiple metastases from a mammary adenocarcinoma, after 150 mg/kg i.v. MIS. ● Plasma MIS; ○ Tumour MIS; ▲ Plasma Ro 05-9963; △ Tumour Ro 05-9963.

for normal brain, of 109 $\mu$g/ml (45% of plasma concentration), was found in Biopsy Specimen 3, which was composed of well differentiated tumour cells and was well vascularized.

*Case* 3 (*mammary adenocarcinoma*). The tumour MIS concentration was maintained at a fairly constant level during the first 3 h and thereafter declined gradually (Fig. 3). The overall mean tumour:plasma concentration ratio was 71 ± 14%.

*Case* 4 (*cutaneous lymphosarcoma*). The maximum tumour concentration was achieved at 1 h and thereafter the concentration steadily declined up to 5 h. The overall mean tumour:plasma concentration ratio was 66 ± 8%.

FIG. 4.—Plasma and tumour nitroimidazole concentrations in a bitch bearing multiple cutaneous lymphosarcomas after 150 mg/kg i.v. MIS. ● Plasma MIS; ○ Tumour MIS; ▲ Plasma Ro 05-9963.

*Case* 5 (*fibrosarcoma*). The tumour MIS concentration was maintained at a steady level for the first 4 h in this case. The overall mean tumour:plasma ratio was 67 ± 6%.

*Case* 6 (*squamous cell carcinoma*). The pattern of MIS concentration in this tumour was somewhat variable, after being maintained at fairly constant level during the first 3 h (Table IVe). The overall mean tumour:plasma concentration ratio was 63 ± 12%.

The concentrations of Ro 05-9963 in the tumour samples were measurable only in the mammary adenocarcinoma, and are recorded in Table IVc. For all other tumour samples the preparation technique (1 vol tumour: 9 vol distilled water) reduced the concentration of the metabolite below 1 μg/ml, and concentrations are therefore recorded as < 10 μg/ml.

In Case 3 the tumour metabolite concentration rose to a maximum at 2 h and was maintained at a fairly steady level up to 5. The overall mean tumour:plasma concentration ratio was high (97 ± 19%), values at 2 and 3 h showing more than complete penetration by the metabolite.

### DISCUSSION

We have studied the concentrations of MIS and its metabolite, Ro 05-9963, in 6 spontaneous canine tumours after doses of 150 mg/kg i.v. in order to investigate the relationship between tumour and plasma concentrations and to elucidate the optimum timing for the irradiation of tumours after the administration of MIS.

Because of the necessity for general anaesthesia in the 4 dogs from which serial biopsy specimens were removed, we have also studied the effect of the simultaneous administration of sodium pentobarbitone on the pharmacokinetics of MIS in 2 dogs. These findings may be compared with the data previously reported by White *et al.* (1979) for the pharmacokinetics of MIS in unanaesthetized dogs.

After sodium pentobarbitone anaesthesia, the pattern of MIS elimination from the plasma was not markedly altered. Peak plasma concentrations occurred at about the same time, and the half-life of MIS remained within the previously reported range (4·9–6·7 h as compared with a range of 3·2–6·9 h). Some differences were noted, however; firstly, the peak plasma MIS concentrations were raised after anaesthesia by 11–13% to values of 200 and 223 μg/ml. However, these values were within the range previously found (172–224 μg/ml) at the dose of 150 mg/kg i.v. (White *et al.*, 1979). Secondly, as a result of the raised peak plasma values the total AUC after anaesthesia was raised by 23 and 34% respectively, to 1942 and 2489 μg/ml.h. These values were rather higher than the previously reported range for this dose (1357–1740 μg/ml.h). Thirdly, the metabolism of MIS to the O-demethylated metabolite, Ro 05-9963, appeared to be delayed by barbiturate anaesthesia, reducing and delaying peak plasma concentrations. The total availability of the metabolite, however, remained unaltered.

The delayed metabolism of MIS, which may well be due to competition with the barbiturate for common metabolizing enzymes, probably accounts for the early high MIS concentrations observed in the 2 anaesthetized dogs.

The above findings suggest that peak plasma MIS concentrations would be somewhat higher in the dogs anaesthetized for the purpose of biopsy. This was in fact the case, and all anaesthetized dogs showed peak plasma concentrations at the upper limit of, or greater than, the normal range for this dose (184–225 μg/ml).

Tumour penetration was good in all cases in this study except Case 2 (meningioma), with values ranging between 47 and 95% of the plasma concentration. In Cases 3–6 the maximum tumour concentration was achieved at 1 h, high concentrations being maintained during the first 3 h after drug administration.

MIS appears suitable as a radiosensitizing agent for veterinary radiotherapy, rapid and high concentrations being achieved in tumours after i.v. dosage, and the timing of radiotherapy not critical within the first 3 h after dosage. The range of maximum tumour concentrations achieved in this study again excepting Case 2 (131–198 μg/ml) indicate that enhancement ratios in the range of 1·8–2·0 might be expected for the response of tumours at this dose level (Dische *et al.*, 1977).

We have also investigated the concentrations of MIS and Ro 05-9963 achieved in the cerebrospinal fluid of the anaesthetized dogs, since the penetration of CSF may well be an important consideration in predicting the neurotoxicity of the drug and also for the timing of radiotherapy for brain tumours in man. Little data are available for the penetration of CSF by MIS. However, Urtasun *et al.* (1977) recorded a level of 82% in a sample taken by lumbar puncture 5·5 h after a dose of 4 g in a patient with a brain tumour. Lu *et al.* (1978) state simply that the plasma and CSF concentrations are identical during the drug's elimination phase in the dog.

The CSF concentrations recorded in this study indicated good penetration by the drug, almost complete penetration occurring at 3 h in both dogs. This was notably later than the time of peak penetration in the tumour in Cases 2–6. MIS appeared able to pass into the CSF freely during the period studied, and ranges of 81–97% and 46–95% were recorded. The metabolite, Ro 05-9963, appeared to pass into the CSF less well, and concentrations ranged between only 15–58% and 0–43% of the plasma concentrations respectively.

Despite the high degree of penetration recorded in the CSF (112%) of Case 2 (meningioma) it appears the dog's brain is only moderately well penetrated by MIS at 3·5 h. The mean concentration found in the brain samples was $114 \pm 9$ μg/ml ($47 \pm 3$% of the plasma concentration). Although there was little variation between the concentrations found in the normal brain, considerable differences were noted between the tumour samples. In the well differentiated and vascular area of Biopsy Specimen 3 the concentration was similar to that elsewhere in normal brain (45%), whilst in the poorly differentiated and relatively avascular areas of Biopsy Specimens 1 and 2 the concentrations represented only 17 and 34% of the plasma concentration. It is debatable whether these variations were attributable to the histological and vascular patterns.

The estimation of the concentration of MIS in tumours will be a valuable guide to the correct timing of radiotherapy to achieve the maximum enhancement of the tumour response, and also in evaluating the likely degree of enhancement. It is important to establish what relationship, if any, exists between tumour and plasma MIS concentrations.

The mouse has been regarded as a poor species in which to investigate such a relationship, since it has generally been held that the short half-life of MIS in the mouse has led to poor tumour concentrations and a lower tumour: plasma ratio than in man. Dische *et al.* (1977) reported concentrations of less than 40% of the plasma concentration in mouse tumours, and similar levels have been recorded elsewhere (McNally *et al.*, 1978).

Generally higher levels were reported in man by Gray *et al.* (1976) but considerable variation was recorded between tumours (12–92%). Subsequent reports showed less variation, but the same high degree of penetration: 37–107% (Dische *et al.*, 1977), 50–70% (Wiltshire *et al.*, 1978) and 50–100% (Workman *et al.*, 1978*b*).

With the exception of Case 2 (meningioma) the range of penetration values in this study (47–95%) closely resembles the range previously reported in man. The mean values for the tumour: plasma ratios for these cases were remarkably constant (69, 71, 66, 67 and 63%) with only small degrees of variation, suggesting that tumour concentrations were largely a function of the available plasma concentrations.

The assumption that the higher tumour penetration values seen in man have been the result of the relatively long half-life of MIS in man has recently been challenged by Brown *et al.* (1979), who recorded tumour: plasma ratios ranging from 50 to 70% in the mouse, and demonstrated that this level of penetration remained unaltered when the half-life of MIS was artificially prolonged from 1·5 to 10 h by bilateral renal ligation of the mice. These workers concluded that the tumour penetration in mice was in fact similar to that in man and that any variation was likely to be the result of individual tumour type.

With the exception of the brain tumour in this study, the range of tumour penetration values indicated a similar tumour: plasma concentration relationship to both mouse and man. We concur with the conclusion of Brown *et al.* (1979) that the tumour concentration is dependant more upon the available plasma concentration than on the half-life of MIS in the particular species.

Although the mouse may well be a better species for the study of tumour penetration by MIS than at first thought, it may prove a poor model for the investigation of other radiosensitizing drugs which are more hydrophilic than MIS and have shorter half-lives. The intermediate half-life of MIS in the dog indicates that this species will provide a better opportunity for pharmacokinetic studies of such drugs in a physiologically normal model. Furthermore, the incidence of spontaneous tumours in the dog, of varying histological types, some of which closely resemble the situation in man, will allow the investigation of tumour penetration by such radiosensitizing drugs and provide a valuable guide to the concentrations likely to be achieved in tumours in man.

We wish to acknowledge the financial support of the MRC and CRC. The authors wish to thank Mrs Jane Donaldson for her technical assistance, Dr Cecil Treip for histopathological assistance with Case 2 and Miss C. M. Wright for typing.

REFERENCES

Asquith, J. C., Watts, M. E., Patel, K., Smithen, C. E. & Adams, G. E. (1974) Electron Affinic sensitisation. V. Radiosensitisation of hypoxic bacteria and mammalian cells *in vitro* by some nitroimidazoles and nitropyrazoles. *Radiat. Res.*, **60**, 108.

Brown, J. M., Yu, N. Y. & Workman, P. (1979) Pharmacokinetic considerations in testing hypoxic cell radiosensitisers in mouse tumours. *Br. J. Cancer*, **39**, 310.

Chapman, J. E., Reuvers, A. P., Borsa, J., Henderson, J. S. & Milgliore, R. D. (1974) Nitroheterocyclic drugs as selective radiosensitisers of hypoxic mammalian cells. *Cancer Chemotherapy Rep.*, **58**, 559.

Denekamp, J., Michael, B. D. & Harris, S. R. (1974) Hypoxic cell radiosensitisers; comparative tests of some electron-affinic compounds using epidermal cell survival *in vivo*. *Radiat. Res.*, **60**, 119.

Dische, S., Saunders, M. I., Lee, M. E., Adams, G. E. & Flockhart, I. R. (1977) Clinical testing of the radiosensitiser Ro 07-0582. Experience with multiple doses. *Br. J. Cancer*, **35**, 367.

Gray, A. J., Dische, S., Adams, G. E., Flockhart, I. R. & Foster, J. L. (1976) Clinical testing of the radiosensitiser Ro 07-0582. I. Dose tolerance, serum and tumour concentrations. *Clin. Radiol.*, **27,** 151.

LU, K., RAULSTON, G. L., BENNETT, K. R. & LOO, T. L. (1978) Pharmacokinetic studies of the new sensitiser 1- (2-nitro-1-imidazol)-3-methoxy-2-propanol (NIMP). *Proc. Am. Ass. Cancer. Res.*, **19,** 150.

MCNALLY, N. J., DENEKAMP, J., SHELDON, P. W., FLOCKHART, I. R. & STEWART, F. A. (1978) Radiosensitisation by misonidazole (Ro 07-0582). The importance of timing and tumour concentration of sensitizer. *Radiat. Res.*, **73,** 568.

URTASUN, R. C., BAND, P., CHAPMAN, J. D., RABIN, H. R., WILSON, A. F. & FRYER, C. G. (1977) Clinical Phase I study of the hypoxic cell radiosensitiser Ro 07-0582, a 2-nitroimidazole derivative. *Radiology*, **122,** 801.

WHITE, R. A. S., WORKMAN, P., FREEDMAN, L. S., OWEN, L. N. & BLEEHEN, N. M. (1979) The pharmacokinetics of misonidazole in the dog. *Euro. J. Cancer* (in press).

WILTSHIRE, C. R., WORKMAN, P., WATSON, J. V. & BLEEHEN, N. M. (1978) Clinical studies with misonidazole. *Br. J. Cancer*, **37,** Suppl. III, 286.

WORKMAN, P., LITTLE, C. J., MARTEN, T. R. & 4 others (1978*a*) Estimation of the hypoxic cell sensitiser misonidazole and its O-demethylated metabolite in biological materials by reversed-phase high-performance liquid chromatography. *J. Chromatogr.*, **145,** 507.

WORKMAN, P., WILTSHIRE, C. R., PLOWMAN, P. N. & BLEEHEN, N. M. (1978*b*) Monitoring of salivary misonidazole in man: A possible alternative to plasma monitoring. *Br. J. Cancer*, **38,** 709.